# Accuracy of emergency department triage using the Emergency Severity Index and independent predictors of under-triage and over-triage in Brazil: a retrospective cohort analysis

**DOI:** 10.1186/s12245-017-0161-8

**Published:** 2018-01-15

**Authors:** Jeremiah S. Hinson, Diego A. Martinez, Paulo S. K. Schmitz, Matthew Toerper, Danieli Radu, James Scheulen, Sarah A. Stewart de Ramirez, Scott Levin

**Affiliations:** 1 Department of Emergency Medicine, Johns Hopkins University School of Medicine, 801 Smith Avenue, Davis Building, Suite 3220, Baltimore, MD 21209 USA; 2 Department of Operations Integration, Johns Hopkins Hospital, Baltimore, MD USA; 3 Emergency Department, Hospital Moinhos de Vento, Porto Alegre, Brazil; 4 Whiting School of Engineering, Johns Hopkins University, Baltimore, MD USA; 5 Systems Institute, Johns Hopkins University, Baltimore, MD USA

**Keywords:** Triage, Mistriage, Emergency severity index, Emergency department

## Abstract

**Background:**

Emergency department (ED) triage is performed to prioritize care for patients with critical and time-sensitive illness. Triage errors create opportunity for increased morbidity and mortality. Here, we sought to measure the frequency of under- and over-triage of patients by nurses using the Emergency Severity Index (ESI) in Brazil and to identify factors independently associated with each.

**Methods:**

This was a single-center retrospective cohort study. The accuracy of initial ESI score assignment was determined by comparison with a score entered at the close of each ED encounter by treating physicians with full knowledge of actual resource utilization, disposition, and acute outcomes. Chi-square analysis was used to validate this surrogate gold standard, via comparison of associations with disposition and clinical outcomes. Independent predictors of under- and over-triage were identified by multivariate logistic regression.

**Results:**

Initial ESI-determined triage score was classified as inaccurate for 16,426 of 96,071 patient encounters. Under-triage was associated with a significantly higher rate of admission and critical outcome, while over-triage was associated with a lower rate of both. A number of factors identifiable at time of presentation including advanced age, bradycardia, tachycardia, hypoxia, hyperthermia, and several specific chief complaints (i.e., neurologic complaints, chest pain, shortness of breath) were identified as independent predictors of under-triage, while other chief complaints (i.e., hypertension and allergic complaints) were independent predictors of over-triage.

**Conclusions:**

Despite rigorous and ongoing training of ESI users, a large number of patients in this cohort were under- or over-triaged. Advanced age, vital sign derangements, and specific chief complaints—all subject to limited guidance by the ESI algorithm—were particularly under-appreciated.

## Background

The primary objective of triage is to rapidly identify patients with critical and time-sensitive conditions and to prioritize their care above those who can wait [1]. Effective triage is required when demand for medical care outstrips capacity, as has become commonplace in the emergency department (ED) due to overcrowding, now recognized as a major threat to patient safety and quality care across the globe [2–5].

In this context, the accuracy and reliability of ED triage are paramount. Under-triage, or failure to identify and differentiate patients with acutely severe illness (e.g., myocardial ischemia, sepsis) from those with less urgent needs (e.g., indigestion, minor infections), contributes to delays in time-sensitive interventions and to potentially avoidable clinical deterioration, morbidity, and mortality [6–9]. Consequential delays to thrombolytic therapy [10], percutaneous coronary intervention [11], antibiotic administration [12], asthma treatment [13], and analgesic administration [14] have all been associated with ED crowding and place under triaged patients at undue risk. Over-triage, or inappropriate labeling of patients with non-urgent presentations to high acuity designations, may have indirect, but equally harmful effects. Triage level designation is often associated with tracking of patients to specific ED care locations based on anticipated resource need [15], and triage level has been shown to drive physician resource utilization decisions, including hospital admission [16]. Thus, over-triage results in diversion of limited time and resources from patients most in need and inappropriate allocation to those with less severe illness.

The most widely used ED triage tools employ a five-level triage scale and include the Australian Triage Scale [17], Canadian Emergency Department Triage and Acuity Scale (CTAS) [18], Manchester Triage Scale (MTS) [19], and the Emergency Severity Index (ESI) [20]. ESI was developed in the USA and is being adopted by an increasing number of EDs globally [20, 21]. Despite its widespread adoption and numerous strengths that include ease of use and linkage to anticipated ED resource utilization [20], ESI has several limitations. It relies heavily on provider judgment and intuition, allowing for significant practice variation, with inter-rater reliability reported to range from *k* = 0.46 to 0.91 [22]. More than half of all visits in the USA are triaged to ESI level 3, generating a large pool of undifferentiated patients that creates challenges for efficient ED resource distribution and effective patient queuing, undermining the very purpose of triage [23, 24] Furthermore, ESI has never been well-validated against critical outcomes indicating time-sensitive needs in any setting [20].

In this study, we evaluated the performance of ESI in the ED of a large tertiary care academic hospital in Porto Alegre, Brazil, and identified independent predictors of under- and over-triage by nurses using ESI. We also measured the impact of under- and over-triage on hospitalization and critical outcomes.

## Methods

### Study design

We used a single-center retrospective cohort study design to characterize ED triage patterns. Records for all ED visits during the study period were retrieved from a relational database underlying the study institution electronic health record (EHR) by an experienced data user and were de-identified prior to analysis by the study team. This study was approved by the institutional review boards of all participating institutions.

### Study setting and population

All patient visits occurred at a large tertiary care academic hospital in Porto Alegre, Brazil, with Joint Commission International accreditation, an ED census of 78,000 visits per year and a mean annual admission rate of 12%. All ED triage at this institution is performed using ESI, and all study site triage nurses have undergone formal training in the use of ESI, passed ESI competency exams, and receive annual refresher training sessions. Visits for adult patients (≥ 18 years old) who presented for care between January 1, 2013, and September 13, 2015, were included for analysis.

### Measures

#### Triage level designations and gold standard comparator

As a part of routine clinical care, ESI triage levels were assigned by a nurse with formal training in ESI for all patients at the time of ED arrival. Nurse-assigned ESI triage level was used to guide clinical care. For administrative purposes unrelated to this study, a second ESI triage level was entered for all patients at the close of ED encounter by the treating emergency physician. Both nurse and physician triage level designations were made according to the standardized ESI algorithm, but physician ESI level designation was made with full knowledge of actual ED resource utilization and acute clinical outcomes. Physician-assigned ESI triage level (assigned a posteriori) was used as a surrogate gold standard for accurate triage and was validated as such by the measurement of association with hospital admission and composite critical outcome using chi-square analysis (see Fig. 2). Prior to analysis, ESI triage scores were designated as high acuity (ESI level 1 or 2), moderate acuity (ESI level 3), or low acuity (ESI level 4 or 5). Redistribution of triage scores from five to three tiers was performed prior to analysis to more effectively capture the clinical impact of triage decisions, as ESI triage levels 1 and 2 are considered time-sensitive and are roomed immediately, while ESI level 3 patients often wait hours to receive definitive care, and ESI levels 4 and 5 are cared for in a separate area of the ED with a *fast track* designation. Thus, under-triage from ESI level 4 to ESI level 5 (both low acuity and subject to similar clinical care pathways) would be expected to have a much smaller effect on patient care and outcomes than under-triage from ESI level 3 (moderate acuity) to ESI level 4 (low acuity), and redistribution to three tiers (high, moderate, low) allowed us to account for this differential effect.

#### Definitions of under- and over-triage

Any patient assigned to a triage tier of lower acuity on arrival (nurse-assigned) than at close of encounter (physician-assigned) was defined as *under-triaged*. Any patient assigned to a triage tier of higher acuity on arrival than at close of ED encounter was defined as *over-triaged*. Patients with the same designation on arrival and at close of ED encounter were defined as *accurate*.

#### Clinical outcomes

Clinical outcome measures included hospital admission, in-hospital mortality, and multiple critical patient outcomes. Hospital admission was defined as any admission to an inpatient care site. In-hospital mortality was defined as death during the index hospital encounter, irrespective of whether it occurred in the ED or during inpatient hospitalization. Composite critical outcome was defined as meeting one or more of the following criteria within 24 h of ED disposition: admission to an intensive care unit (ICU), emergent procedure in an operating room, cardiac catheterization, endoscopy or bronchoscopy, transfer to an outside acute care facility, or in-hospital mortality.

#### Candidate predictor variables

Patient data collected at the time of ED triage and used as candidate predictor variables for under- and over-triage by multivariate logistic regression analysis (see below) included age, sex, vital signs, patient-reported pain score, chief complaint category, and arrival time. Vital signs, including temperature, systolic blood pressure, heart rate, respiratory rate, and oxygen saturation, were classified as normal or gradations of abnormal according to previously established physiologic cutoffs [25–28]. Pain scores were obtained using a visual analog scale that ranged from 1 (least severe) to 10 (most severe), and scores were further classified as mild (≤ 3), moderate (4–7), or severe (≥ 8). Free-text chief complaints were extracted directly from the electronic health record (EHR), and natural language processing software (Python 2.7 with Natural Language Toolkit 3.0) was used to map complaints to1 of 30 chief complaint categories derived from a schema developed by the Agency for Healthcare Research in Quality as previously described [24, 29].

### Data analysis

Dichotomous and categorical data were displayed as absolute and relative frequencies (in percentages) and continuous data as medians with interquartile ranges (IQRs). Rates of under- and over-triage were calculated as the percentage of visits assigned to a higher or lower triage level on arrival than at close of encounter, respectively. Missing data (i.e., sex, vital signs, pain score, chief complaint) were recorded as *null* and included in logistic regression analysis (below). *Chi-square analysis*: Associations between under-triage or over-triage and hospital admission or composite critical outcome were assessed using Pearson’s chi-squared test with Yates’ continuity correction. *Multivariate Logistic Regression Analysis*: Primary outcome measures included rates of under-triage, over-triage, and concordance (accurate triage). We investigated the extent to which specific patient characteristics (age, sex, temperature, pulse, respiratory rate, systolic pressure, oxygen saturation, patient-reported pain score, chief complaint category, and arrival time) were associated with triage score concordance. Logistic regression models of triage misclassification (over- or under-triage versus concordance) that simultaneously incorporated the several patient characteristics were generated. All analyses were performed in R version 2.14.1 using freely distributed statistical packages.

## Results

**Fig. 1:**
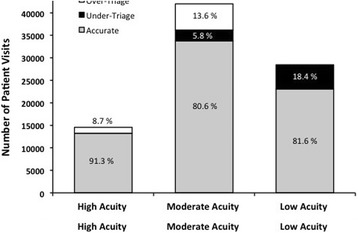
Distribution of ED visits by initial ESI designation and classifications as accurate, over-triage, or under-triage. High acuity = ESI level 1 or 2; moderate acuity = ESI level 3; low acuity = ESI level 4 or 5. ED emergency department; ESI Emergency Severity Index

A total of 96,071 unique adult patient visits were included in our analysis. Patient demographics and clinical characteristics are shown in Table 1. Nearly half of all patient visits (49.1%) were triaged as moderate acuity (ESI level 3) on ED arrival. Roughly one third (32.5%) were triaged as low acuity (ESI level 4 or 5), and only 18.3% were triaged as high acuity (ESI level 1 or 2) (Fig. 1). Using a posteriori physician-assigned triage levels as surrogate gold standard comparators, one fifth of all patients were classified as either under- or over-triaged. For patients triaged to high acuity ESI levels, 8.7% were classified as over-triaged. For patients triaged to moderate acuity on arrival, 13.6% were classified as over-triaged and 5.8% were classified as under-triaged. 18.4% of patients assigned to low acuity ESI levels were classified as under-triaged (Fig. 1).

**Table 1:** Patient demographics and clinical characteristics

Age (IQR)	39 (29.7–59.2)
Sex (%)	
Female	59,091 (61.51)
Male	36,632 (38.13)
Vitals (IQR)	
Temperature (Fahrenheit)	97.3 (96.6–98.2)
Pulse (bpm)	85 (75.0–98.0)
Respiratory rate (rpm)	20 (18.0–20.0)
Systolic blood pressure (mmHg)	128 (117.0–141.0)
Oxygen saturation (%)	98 (97.0–99.0)
Triage level (%)	
ESI 1	272 (0.28)
ESI 2	17,334 (18.04)
ESI 3	47,207 (49.14)
ESI 4	29,921 (31.14)
ESI 5	1337 (1.39)
Arrival time (%)	
8 AM–12:59 PM	30,565 (31.82)
1 PM–4:59 PM	23,085 (24.03)
5 PM–8:59 PM	19,620 (20.42)
9 PM–12:59 AM	12,941 (13.47)
1 AM–7:59 AM	9860 (10.26)
VAS pain score (%)	
Mild (0–3)	39,376 (40.99)
Moderate [4–7]	32,517 (33.85)
Severe [8–10]	22,836 (23.77)
Hospital admission (%)	14,508 (15.10)
Critical outcomes (%)	
ICU admission	183 (0.19)
Death	1019 (1.06)
Surgery	1991 (2.07)
Cardiac catheterization	219 (0.23)
Hospital transfer	48 (0.05)
In-hospital mortality	1290 (1.34)

*bpm* beats per minute, *ICU* intensive care unit, *IQR* interquartile range, *rpm* respirations per minute, *VAS* visual analog scale

### Under-triage and risk for admission or critical outcome

**Fig. 2:**
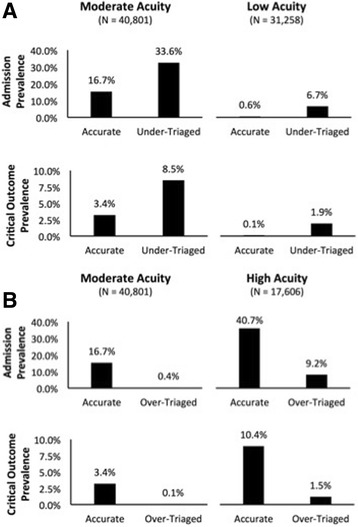
Associations between triage accuracy and clinical outcomes. **a** Under-triage to moderate- and low-acuity ESI triage levels is associated with a significantly increased prevalence of hospital admission and critical outcomes. **b** Over-triage to moderate- and high-acuity ESI triage levels is associated with a significantly decreased prevalence of hospital admission and critical outcomes

As demonstrated in Fig. 2a, patients who were triaged as moderate acuity by ESI (level 3) on arrival but subsequently determined to be more appropriate for high acuity triage level 1 or 2 (i.e., under-triaged) had a significantly increased prevalence of admission and critical outcomes as compared to those appropriately triaged to moderate acuity (*χ*^2^ = 502.06, df = 1, *p* value < 0.001 and *χ*^2^ = 184.91, df = 1, *p* value < 0.001, respectively). Similarly, patients who were under-triaged to low-acuity ESI levels (4 or 5) on arrival had a significantly increased prevalence of admission and critical outcomes as compared to patients appropriately triaged to the same ESI levels (*χ*^2^ = 1033.60, df = 1, *p* value < 0.001 and *χ*^2^ = 343.05, df = 1, *p* value < 0.001, respectively).

### Over-triage and risk for admission or critical outcome

The opposite trend was observed for patients who were over-triaged (Fig. 2b). Those triaged as moderate acuity by ESI on arrival but subsequently determined to be more appropriate for low-acuity triage level 4 or 5 (i.e., over-triaged) had a significantly lower prevalence of admission and critical outcomes as compared to those appropriately triaged to moderate acuity (*χ*^2^ = 1184.90 df = 1, *p* value < 0.001 and *χ*^2^ = 213.04, df = 1, *p* value < 0.001, respectively). Similarly, patients who were over-triaged to high-acuity ESI levels (1 or 2) on arrival had a significantly lower prevalence of admission and critical outcomes as compared to patients appropriately triaged to the same ESI levels (*χ*^2^ = 588.49, df = 1, *p* value < 0.001 and *χ*^2^ = 126.57, df = 1, *p* value < 0.001, respectively).

### Factors associated with over-triage and under-triage

#### Age

Advancing age was associated with under-triage by ESI (Table 2). Odds of under-triage increased in parallel with age and were greatest for patients aged ≥ 70 years (OR 1.50, 95% CI 1.30–1.74 for under-triage to moderate acuity designations and OR 2.2, 95% CI 1.24–3.75 for under-triage to low acuity designations). Conversely, advanced age was associated with low likelihood of over-triage to high or moderate ESI acuity levels (Table 3).

**Table 2:** Factors predictive of under-triage to moderate and low ESI acuity levels^†^

	Moderate acuity (ESI 3)	Low acuity (ESI 4 or 5)
	*N* = 40,801	*N* = 31,258
	Odds ratio^‡^ (95% CI)	Odds ratio^§^ (95% CI)
Age (18–29 years comparator)		
30–49 years	1.25 (1.11–1.41)***	1.15 (1.08–1.22)**
50–69 years	1.48 (1.3–1.69)***	1.48 (1.33–1.63)**
≥ 70 years	1.50 (1.3–1.74)***	2.20 (1.24–3.75)*
Sex (male comparator)		
Female	0.81 (0.75–0.88)***	1.01 (0.95–1.08)
Null (missing)	0.21 (0.01–0.95)	0.02 (0.00–0.07)**
Temperature (normal comparator)		
Hypothermia (≤ 94.0 °F)	1.15 (0.95–1.39)	0.9 (0.73–1.1)
Mild hypothermia (94.1–96.2 °F)	1.01 (0.9–1.13)	0.97 (0.88–1.07)
Mild hyperthermia (99.3–100.4 °F)	0.90 (0.73–1.08)	1.49 (1.33–1.66)**
Hyperthermic (≥ 100.5 °F)	0.98 (0.77–1.24)	1.65 (1.42–1.9)**
Null (missing)	1.41 (1.00–1.94)*	1.42 (1.06–1.88)
Heart rate (normal comparator)		
Severe bradycardia (≤ 49 bpm)	2.55 (1.54–4.02)***	0.61 (0.14–1.82)
Mild bradycardia (50–59 bpm)	1.22 (0.99–1.50)	1.09 (0.85–1.37)
Mild tachycardia (105–109 bpm)	1.08 (0.88–1.32)	1.2 (1.05–1.37)*
Moderate tachycardia (110–119 bpm)	1.13 (0.94–1.34)	1.1 (0.98–1.24)
High tachycardia (120–129 bpm)	1.32 (1.01–1.7)*	1.24 (1.03–1.48)
Severe tachycardia (≥ 130 bpm)	2.17 (1.61–2.88)***	1.46 (1.1–1.93)*
Null (missing)	–	1.78 (0.24–12.12)
Systolic blood pressure (normal comparator)		
Hypotension (≤ 99 mmHg)	0.84 (0.70–0.99)*	1.05 (0.89–1.23)
Mild hypotension (100–107 mmHg)	1.07 (0.88–1.29)	1.15 (1.03–1.28)*
Mild hypertension (177–199 mmHg)	1.26 (1.03–1.52)*	1.01 (0.6–1.63)
Hypertension (≥ 200 mmHg)	1.34 (0.78–2.16)	0.78 (0.26–1.88)
Null (missing)	0.55 (0.23–1.17)	1.65 (1.03–2.56)
Respiratory rate (normal comparator)		
Hypopnea (≤ 13 rpm)	1.59 (0.65–3.29)	0.78 (0.4–1.39)
Mild hypopnea (14–15 rpm)	1.19 (1.00–1.41)*	1.04 (0.92–1.16)
Mild tachypnea (20–22 rpm)	0.9 (0.81–0.99)*	1.07 (0.99–1.16)
Moderate tachypnea (23–27 rpm)	0.83 (0.68–1.00)	1.23 (1.02–1.49)
Severe tachypnea (≥ 28 rpm)	0.99 (0.56–1.62)	1.38 (0.78–2.36)
Null (missing)	0.57 (0.06–4.02)	0.14 (0.01–1.24)
Oxygen saturation (normal comparator)		
Severe hypoxia (SpO2 ≤ 89)	2.18 (1.48–3.12)***	1.48 (0.84–2.5)
Moderate hypoxia (SpO2 90–94)	1.12 (0.97–1.28)	1.10 (0.94–1.29)
Mild hypoxia (SpO2 95–96)	1.13 (1.00–1.27)*	1.05 (0.95–1.17)
Null (missing)	2.81 (1.23–5.74)**	2.10 (0.76–5.24)
Pain score (VAS, moderate comparator)		
Mild (0–3)	0.81 (0.73–0.9)***	0.83 (0.77–0.88)**
Severe [8–10]	1.23 (1.11–1.36)***	1.11 (1.01–1.22)
Null (missing or > 10)	0.47 (0.17–1.04)	0.42 (0.11–1.17)
Chief complaint (general comparator)		
Abdominal pain	1.13 (0.97–1.31)	1.8 (1.57–2.06)**
Allergic	0.49 (0.27–0.81)*	0.42 (0.30–0.57)**
Altered mental status	1.55 (0.98–2.36)	1.74 (0.79–3.56)
Back pain	1.01 (0.81–1.26)	1.47 (1.19–1.82)**
Chest pain	1.43 (1.17–1.73)***	1.88 (1.54–2.29)**
Dizziness	0.98 (0.79–1.22)	0.91 (0.74–1.11)
Dysrhythmia	1.23 (0.79–1.84)	1.22 (0.65–2.14)
Edema	0.44 (0.29–0.64)***	0.71 (0.54–0.93)
Ear, nose, and throat	0.88 (0.47–1.50)	0.27 (0.18–0.38)**
Fever	0.61 (0.48–0.77)***	0.83 (0.72–0.95)*
Genitourinary	0.69 (0.52–0.9)**	0.95 (0.79–1.14)
Gastrointestinal bleeding	0.68 (0.36–1.19)	0.73 (0.31–1.48)
Headache	0.82 (0.67–1.01)	0.75 (0.63–0.88)**
Hypertension	0.60 (0.28–1.13)	0.92 (0.46–1.69)
Lower respiratory tract infection	0.36 (0.26–0.47)***	0.76 (0.66–0.88)**
Musculoskeletal (atraumatic)	0.48 (0.27–0.79)**	0.31 (0.22–0.43)**
Musculoskeletal (isolated trauma)	0.39 (0.12–0.94)	0.36 (0.09–1.03)
Neurologic	1.48 (1.17–1.87)***	1.18 (0.82–1.67)
Nausea, vomiting, and diarrhea	0.57 (0.44–0.73)***	0.84 (0.71–0.99)
Seizure	0.78 (0.47–1.22)	1.65 (1.08–2.48)
Shortness of breath	1.06 (0.85–1.32)	1.49 (1.25–1.78)**
Skin, nails, and hair	0.30 (0.15–0.55)***	0.28 (0.2–0.38)**
Substance abuse	0.81 (0.24–1.99)	0.59 (0.14–1.76)
Syncope	1.41 (1.03–1.90)*	1.68 (0.96–2.84)
Trauma	0.77 (0.59–1.00)	0.70 (0.52–0.92)
Upper respiratory tract infection	0.44 (0.24–0.75)**	0.26 (0.21–0.32)**
Weakness	0.78 (0.57–1.05)	0.96 (0.76–1.21)
Wound	0.35 (0.16–0.66)**	0.45 (0.28–0.68)**
Other	0.58 (0.39–0.84)**	0.51 (0.38–0.67)**
Null (missing)	–	0.68 (0.57–0.81)**
Arrival time (17:00–20:59 comparator)		
01:00–07:59	1.23 (1.06–1.44)**	1.77 (1.58–1.99)**
08:00–12:59	1.48 (1.32–1.67)***	1.75 (1.60–1.90)**
13:00–16:59	1.20 (1.06–1.36)**	1.37 (1.24–1.50)**
21:00–00:59	0.91 (0.78–1.06)	1.04 (0.92–1.17)

^†^Results are odds ratios with 95% confidence intervals in parentheses

*bpm* beats per minute, *rpm* respirations per minute, *VAS* visual analog scale

^‡^Odds ratio of being assigned ESI triage level 3 on arrival and subsequently being determined more appropriate for ESI level 1 or 2

^§^Odds ratio of being assigned ESI triage level 4 or 5 on arrival and subsequently being determined more appropriate for ESI level 1, 2, or 3

*Significant at the 5% level

**Significant at the 1% level

***Significant at the 0.1% level

**Table 3:** Factors predictive of over-triage to high and moderate ESI acuity levels^†^

	High acuity (ESI 1 or 2)	Moderate acuity (ESI 3)
	*N* = 17,606	*N* = 44,470
	Odds ratio^‡^ (95% CI)	Odds ratio^§^ (95% CI)
Age (18–29 years comparator)		
30–49 years	0.80 (0.68, 0.93)**	0.79 (0.73,0.84)***
50–69 years	0.62 (0.52, 0.74)***	0.65 (0.6–0.71)***
≥ 70 years	0.37 (0.31, 0.46)***	0.42 (0.37–0.46)***
Sex (male comparator)		
Female	1.40 (1.24–1.57)***	1.12 (1.06–1.19)***
Null (missing)	–	0.68 (0.26–1.49)
Temperature (normal comparator)		
Hypothermia (≤ 94.0 °F)	0.80 (0.63, 1.01)	1.05 (0.97–1.14)
Mild hypothermia (94.1–96.2 °F)	1.00 (0.86, 1.17)	1.03 (0.89–1.19)
Mild hyperthermia (99.3–100.4 °F)	0.99 (0.73, 1.33)	0.81 (0.71–0.92)**
Hyperthermic (≥ 100.5 °F)	1.28 (0.89, 1.79)	0.66 (0.54–0.79)***
Null (missing)	0.68 (0.40, 1.09)	0.93 (0.71–1.19)
Heart rate (normal comparator)		
Severe bradycardia (≤ 49 bpm)	0.46 (0.19, 0.91)*	0.75 (0.39–1.31)
Mild bradycardia (50–59 bpm)	1.11 (0.83, 1.46)	0.99 (0.83–1.17)
Mild tachycardia (105–109 bpm)	0.83 (0.61, 1.11)	0.89 (0.78–1.03)
Moderate tachycardia (110–119 bpm)	0.84 (0.66, 1.05)	0.97 (0.86–1.09)
High tachycardia (120–129 bpm)	0.83 (0.61, 1.11)	0.94 (0.78–1.13)
Severe tachycardia (≥ 130 bpm)	0.35 (0.23, 0.51)***	0.88 (0.67–1.14)
Null (missing)	0.49 (0.05, 4.48)	1.23 (0.22–6.74)
Systolic blood pressure (normal comparator)		
Hypotension (≤ 99 mmHg)	0.71 (0.55, 0.91)**	0.85 (0.73–0.97)*
Mild hypotension (100–107 mmHg)	1.04 (0.82, 1.3)	0.95 (0.86–1.06)
Mild hypertension (177–199 mmHg)	0.94 (0.75, 1.17)	0.78 (0.65–0.92)**
Hypertension (≥ 200 mmHg)	1.02 (0.75, 1.38)	0.99 (0.63–1.48)
Null (missing)	1.89 (1.04, 3.22)*	–
Respiratory rate (normal comparator)		
Hypopnea (≤ 13 rpm)	0.00 (0.00, 0.01)	1.11 (0.56–2.01)
Mild hypopnea (14–15 rpm)	0.77 (0.57, 1.03)	1.04 (0.92–1.18)
Mild tachypnea (20–22 rpm)	1.05 (0.92, 1.19)	1.04 (0.97–1.11)
Moderate tachypnea (23–27 rpm)	0.96 (0.78, 1.17)	0.87 (0.76–1.00)*
Severe tachypnea (≥ 28 rpm)	0.75 (0.47, 1.14)	0.79 (0.49–1.22)
Null (missing)	2.84 (0.35, 14.23)	0.80 (0.11–4.28)
Oxygen saturation (normal comparator)		
Severe hypoxia (SpO2 ≤ 89)	0.40 (0.25, 0.62)***	0.85 (0.55–1.25)
Moderate hypoxia (SpO2 90–94)	0.72 (0.59, 0.88)**	0.62 (0.55–0.7)***
Mild hypoxia (SpO2 95–96)	0.81 (0.67, 0.97)*	0.86 (0.78–0.94)***
Null (missing)	0.31 (0.05, 1.2)	0.67 (0.21–1.68)
Pain score (VAS, moderate comparator)		
Mild (0–3)	0.80 (0.68, 0.95)*	0.86 (0.78–0.94)***
Severe [8–10]	0.79 (0.68, 0.93)**	0.85 (0.55–1.25)
Null (missing or > 10)	0.23 (0.06, 0.67)*	0.67 (0.21–1.68)
Chief complaint (general comparator)		
Abdominal pain	0.84 (0.67, 1.06)	0.77 (0.69–0.87)***
Allergic	2.14 (1.33, 3.34)**	1.96 (1.57–2.45)***
Altered mental status	0.63 (0.30, 1.17)	0.36 (0.16–0.69)**
Back pain	0.83 (0.61, 1.11)	1.08 (0.91–1.27)
Chest pain	1.04 (0.78, 1.4)	0.68 (0.57–0.81)***
Dizziness	1.36 (0.97, 1.89)	1.13 (0.96–1.32)
Dysrhythmia	1.31 (0.72, 2.24)	1.00 (0.72–1.38)
Edema	1.33 (0.7, 2.33)	1.37 (1.12–1.67)**
Ear, nose, and throat	1.67 (0.88, 2.97)	2.67 (1.99–3.55)***
Fever	1.52 (1.01, 2.25)*	0.94 (0.81–1.10)
Genitourinary	1.23 (0.81, 1.81)	1.08 (0.90–1.28)
Gastrointestinal bleeding	1.04 (0.53, 1.88)	1.06 (0.73–1.51)
Headache	1.21 (0.92, 1.58)	1.38 (1.21–1.58)***
Hypertension	2.00 (1.02, 3.67)*	1.88 (1.29–2.66)***
Lower respiratory tract infection	2.15 (1.28, 3.49)**	1.43 (1.23–1.66)***
Musculoskeletal (atraumatic)	1.62 (0.83, 2.92)	2.21 (1.76–2.77)***
Musculoskeletal (isolated trauma)	1.02 (0.24, 2.91)	0.70 (0.36–1.24)
Neurologic	1.13 (0.84, 1.52)	0.79 (0.64–0.96)*
Nausea, vomiting, and diarrhea	1.37 (0.99, 1.88)	1.33 (1.15–1.54)***
Seizure	0.85 (0.48, 1.41)	0.77 (0.53–1.08)
Shortness of breath	1.33 (0.96, 1.82)	0.89 (0.75–1.06)
Skin, nails, and hair	1.65 (0.77, 3.21)	2.32 (1.88–2.87)***
Substance abuse	2.11 (0.76, 4.97)	0.45 (0.14–1.11)
Syncope	0.69 (0.45, 1.03)	0.75 (0.56–0.98)*
Trauma	0.92 (0.65, 1.30)	0.89 (0.73–1.07)
Upper respiratory tract infection	2.11 (0.70, 5.24)	2.82 (2.27–3.5)***
Weakness	1.71 (1.13, 2.54)**	1.36 (1.12–1.65)**
Wound	0.64 (0.22, 1.48)	1.58 (1.2–2.06)***
Other	0.94 (0.59, 1.44)	1.39 (1.14–1.69)**
Null (missing)	1.02 (0.79, 1.31)	1.00 (0.87–1.14)
Arrival time (17:00–20:59 comparator)		
01:00–07:59	1.02 (0.83, 1.25)	0.84 (0.76–0.94)**
08:00–12:59	1.37 (1.17, 1.61)***	1.23 (1.14–1.33)***
13:00–16:59	1.21 (1.02, 1.43)*	1.07 (0.99–1.16)
21:00–00:59	0.99 (0.82, 1.19)	0.76 (0.69–0.84)***

^†^Results are odds ratios with 95% confidence intervals in parentheses

*bpm* beats per minute, *rpm* respirations per minute, *VAS* visual analog scale

^‡^Odds ratio of being assigned ESI triage level 1 or 2 on arrival and subsequently being determined more appropriate for ESI level 3, 4, or 5

^§^Odds ratio of being assigned ESI triage level 3 on arrival and subsequently being determined more appropriate for ESI level 4 or 5

*Significant at the 5% level

**Significant at the 1% level

***Significant at the 0.1% level

#### Vital signs

There were several associations between triage vital signs and under-triage using ESI. Most notably, severe bradycardia, tachycardia, and hypoxia were all strongly associated with under-triage of high-acuity patients to moderate-acuity ESI triage levels (OR 2.55, 95% CI 1.54–4.02; OR 2.17, 95% CI 1.61–2.88; and OR 2.18, 95% CI 1.48–3.12, respectively). Importantly, the absence of oxygen saturation measurements was also strongly associated with under-triage to moderate acuity (OR 2.81, 95% CI 1.23–5.74). Mild abnormalities in vital signs were more likely to be associated with under-triage to low-acuity ESI levels, including mild tachycardia (OR 1.20, 95% CI 1.05–1.37) and mild hypotension (OR 1.15, 95% CI 1.03–1.28). An exception was temperature, where patients with both borderline and frank hyperthermia were both more likely to be under-triaged to low-acuity ESI triage levels (OR 1.49, 95% CI 1.33–1.66 and OR 1.65, 95% CI 1.42–1.90, respectively). There were no vital sign abnormalities associated with increased likelihood of over-triage to any acuity level (Table 3).

#### Chief complaints

A number of chief complaints were associated with under-triage (Table 3). Those most likely to result in a triage of high-acuity patients to moderate-acuity ESI levels included neurologic complaints (OR 1.48, 95% CI 1.17–1.87), syncope (OR 1.41, 95% CI 1.03–1.90), and chest pain (OR 1.43, 95% CI 1.17–1.73). Complaints most likely to be under-triaged to low-acuity ESI levels were chest pain (OR 1.88, 95% CI 1.54–2.29) and abdominal pain (1.80, 95% CI 1.57–2.06), followed by shortness of breath (OR 1.49, 95% CI 1.25–1.78) and back pain (OR 1.47, 95% CI 1.19–1.82). On the converse, strongest associations with over-triage of low-acuity patients to moderate-acuity triage levels were those related to upper respiratory tract infections (OR 2.82, 95% CI 2.27–3.50); ear, nose, and throat (ENT) complaints (OR 2.67, 95% CI 1.99–3.55); skin, nails, and hair complaints (OR 2.32, 95% CI 1.88–2.87); allergic complaints (OR 1.96, 95% CI 1.57–2.45); and hypertension (OR 1.88, 95% CI 1.29–2.66). Lower respiratory tract infections, allergic complaints, and hypertension were most strongly associated with over-triage to high-acuity ESI levels (OR 2.15, 95% CI 1.28–3.49; OR 2.14, 95% CI 1.33–3.34; and OR 2.00, 95% CI 1.02–3.67, respectively).

## Discussion

In this study, we evaluated the predictive accuracy of ED triage by nurses applying ESI using a novel approach that leveraged both clinical judgment of a treating physician with knowledge of actual ED resource utilization and clinical outcome data captured from the EHR. We found that nearly one in five patients was under- or over-triaged by ESI on ED arrival and that this was caused by both under-recognition of high acuity clinical presentations and overestimation of urgency in patients without severe illness. Importantly, we found that under-triage was associated with increased hospital admission rates and greater likelihood for critical clinical outcomes. These findings are consistent with prior findings that failure to distinguish patients with critical and time-sensitive conditions contributes to the delays in disposition and time-sensitive treatments and to the increases in potentially avoidable morbidity and mortality [6–9]. Partly as a result of mistriage, approximately one half of all patients in this population were designated with the same triage score [3] using this five-level index. A similar lack of patient differentiation has also been observed widely in the USA and has been cited as a specific limitation of ESI [24, 30].

Perhaps most importantly, this study identified many factors highly predictive of under- and over-triage by ESI, several of which related to objective data routinely collected on ED arrival. Advanced age was strongly associated with under-triage, suggesting the impact of patient age on initial presentation and clinical course is not well-recognized by ESI and under-appreciated by triage clinicians. While the ESI training manual suggests consideration of age when determining whether a particular presentation is a high risk, no specific recommendations or guidelines related to age are specified [20] and ESI has previously been shown to have poor sensitivity for identification of elderly patients requiring life-saving interventions [31].

Similarly, there was a strong association between multiple vital sign abnormalities (bradycardia, tachycardia, hypotension, hypoxia, and hyperthermia) and under-triage, again suggesting under-appreciation by ESI. It has been well-established that abnormalities in triage vital signs are strong predictors of adverse outcomes including ICU admission and in-hospital mortality [25]. Specific recommendations related to vital signs are provided by ESI, with a suggestion to consider increasing the acuity level assignment for patients with “danger zone vitals,” defined as tachycardia (heart rate > 100 bpm), tachypnea (respiratory rate > 20 rpm), or hypoxia (SpO2 < 92%) [20]. No specific recommendations are provided for patients with bradycardia or derangements in blood pressure or temperature. Good correlation between abnormal vital signs and ESI acuity level assignment was previously reported, but authors of this study utilized an aggregate vital sign score for analysis and the finding that abnormal vital signs are, in general, more likely to fall into higher acuity triage levels should not be surprising [32]. On the converse, our study demonstrates that subtle vital sign abnormalities are systematically under-appreciated by ESI that vital sign derangements for which there are no recommendations provided by ESI are under-recognized and associated with mistriage.

Last, we found strong associations between several chief complaint categories and mistriage. Many of these are understood intuitively. For example, strongest associations with over-triage were seen for allergic reactions, ENT complaints, and respiratory infections (Table 3). Worst-case scenarios for patients in each of these categories involve compromise of airway and breathing. However, the actual severity of illness may not be readily apparent to the triage provider who is without access to a full exam and diagnostics. Under a system that relies heavily on nursing judgment, it is easy to understand how such patients might be triaged to acuity levels higher than ultimately needed. Other associations, however, are more surprising. Chest pain and shortness of breath, for example, are complaints strongly associated with high morbidity conditions including acute coronary syndrome and pulmonary embolism [33, 34], yet both were associated with *under-triage* in this study. While some standardized triage scales including CTAS [18] and MTS [19] utilize standardized lists of presenting complaints or symptoms in triage score assignment [18, 19], they are not addressed directly in the ESI algorithm. Instead, nurses are instructed to consider the presenting complaint in the entire context of the patient when assigning a triage score [20], generating a potential for high variability among users.

While our findings suggest several potential weaknesses of ESI, the scale remains an extremely popular triage tool with many strengths. The simplistic algorithm by which ESI scores are assigned allows nurses to make rapid triage decisions by answering only three questions: (1) Is the patient dying? (2) Should the patient wait? and (3) How many resources will this patient require? [20] While the answers to these questions are certainly influenced by additional factors including patient appearance, chief complaint, and vital signs, a level of autonomy is maintained by the triage provider using ESI that allows for increased efficiency and ease of use. The simplicity of the ESI algorithm is also an important operational strength, as the algorithm can be memorized easily and there is little need for cumbersome paper or electronic reference material in the clinical environment. Finally, the incorporation of resource utilization prediction by ESI is unique among ED triage tools and makes it particularly useful in limited resource settings.

### Limitations

This study is strengthened by its large sample size and the inclusion of an a posteriori triage score assignment with EHR-based clinical outcome validation as comparator, yet several limitations of our findings should be considered. ESI aims to assign patients to triage categories based on both severity of disease presentation and anticipated ED resource utilization. While all physicians who assigned a posteriori ESI scores had full knowledge of actual ED resource utilization, the retrospective nature of our work limits our ability to confirm that these scores accurately reflect resource utilization. However, ED resource utilization has been shown to correlate well with disposition and clinical outcomes, and our findings that patients deemed under-triaged were more likely to be hospitalized or experience critical events supports the validity of our comparator [24, 35, 36]. Additionally, all patient encounters occurred at a single institution where ED nurses undergo structured and ongoing training in ESI triage. It is possible that both distribution of triage score assignment and clinical course at other sites may differ. Indeed, it is likely that in settings where ESI has been adopted for clinical use without implementation of structured training programs for ED nurses, under- and over-triage would be more frequent than observed here and our findings may represent an overestimate of ESI performance in general.

## Conclusions

ESI has many strengths and is among the most widely used of all standardized triage tools, yet a large number of patients in our cohort were under- and over-triaged using this scale. Despite rigorous training of triage providers, reliance on human experience and intuition under the ESI algorithm allowed for under-appreciation of the clinical impact of age, subtle vital sign abnormalities, and multiple specific complaints. These findings should be used to inform ESI users, ESI training programs, and future iterations of ESI. Perhaps more importantly, these findings provide a rationale for the development of future triage tools that are both efficient and objective.
